# Supplementary material for: Macromolecular and electrical coupling between inner hair cells in the rodent cochlea
Source: Nat Commun. 2020 Jun 25;11:3208. doi: 10.1038/s41467-020-17003-z (PMC7316811; doi:10.1038/s41467-020-17003-z)
Supplement: Supplementary file 3 — Description of Additional Supplementary Files [file 41467_2020_17003_MOESM3_ESM.pdf]

Description of Additional Supplementary Files

File Name: Supplementary Movie 1

Description: Macromolecular coupling between two IHCs. Diffusion of the fluorescent TAMRA-peptide pipette-loaded in one IHC to the neighboring IHC.

File Name: Supplementary Movie 2

Description: Z-stack of five coupled IHCs. Z-stack of TAMRA-peptide fluorescence from bottom to top of a mini-syncytia of five coupled IHCs. The patched IHC can be recognized by a hole at its basolateral membrane.

File Name: Supplementary Movie 3

Description: 3D-view of five coupled IHCs. 3D-view of TAMRA-peptide fluorescence in a mini-syncytia of five coupled IHCs. The patched IHC can be recognized by a hole at its basolateral membrane.

File Name: Supplementary Movie 4

Description: 3D reconstruction of FIB-SEM image stack of neighboring IHCs (P34) illustrating a flat membrane contact. 3D reconstruction shows the neighboring membranes (highlighted in blue and red) forming flat contacts.

File Name: Supplementary Movie 5

Description: Sequential FIB-SEM images and the 3D reconstruction of P34 IHCs revealing a putative membrane fusion site. Illustrated are neighbouring membranes (highlighted as blue and red 3D reconstructions) of two basolateral IHCs that form a contact leading to a putative fusion site.

File Name: Supplementary Movie 6

Description: Lack of physical contacts between outer hair cells. 3D reconstruction of serial block-face electron microscopic datasets demonstrates that the basolateral membranes of P22 mouse midcochlear outer hair cells (in blue) do not share physical contacts between each other. Outer hair cells and surrounding supporting cells are initially showed in yellow. Additional oblique slices of the used datasets are demonstrated in the supplemental figure 9.

File Name: Supplementary Movie 7

Description: Live calcium imaging of two coupled IHCs. Depicted are the calcium hotspots (indicated by arrows) that appear in the two coupled IHCs upon membrane depolarization. Bottom, middle and top sections of the IHCs are showed.
